# Solution to the problem of bridge structure damage identification by a response surface method and an imperialist competitive algorithm

**DOI:** 10.1038/s41598-022-17457-9

**Published:** 2022-10-03

**Authors:** Dan Ye, Zhe Xu, Yangqing Liu

**Affiliations:** 1 Chongqing University of Education, Chongqing, 400065 China; 2 State Key Laboratory of Mountain Bridge and Tunnel Engineering, Chongqing Jiaotong University, Chongqing, 400074 China; 3 Guangxi Communications Investiment Group Co., Ltd., Nanning, 530022 China

**Keywords:** Civil engineering, Scientific data

## Abstract

To increase the efficiency of structural damage identification (SDI) methods and timeously and accurately detect initial structural damage, this research develops an SDI method based on a response surface method (RSM) and an imperialist competitive algorithm (ICA). At first, a Latin hypercube design method is used for experimental design and selection of sample points based on RSM. Then, a high-order response surface surrogate model for the target frequency response and stiffness reduction factor is established. Finally, analysis of variance is performed to assess the overall goodness-of-fit and prediction accuracy of the established model. Then the results obtained are combined with structural dynamic response data to construct objective functions; furthermore, the optimal solution of parameter vector in the objective function is solved based on the ICA. Then damage positioning and quantification can be achieved according to location and degree of change in each parameter; finally, the RSM-ICA-based SDI method proposed is applied to damage identification of high-dimensional damaged simply-supported beam models. To verify the effectiveness of the proposed method, the damage identification results are compared with the results obtained from traditional optimization algorithms. The results indicate that: average errors in the structural stiffness parameters and natural frequency that are identified by the proposed method are 6.104% and 0.134% respectively. The RSM-ICA-based SDI method can more accurately identify the location and degree of damages with more significantly increased identification efficiency and better precision compared to traditional algorithms. This approach provides a novel means of solving SDI problems.

## Introduction

With the increase in the operation time of building structures, the mechanical properties of structures after being put into use are deteriorating because of the coupling effect of harsh service environment, degradation of internal materials, and complex load, which even threaten the safety of the whole structural system^1^. Hence, how to timeously and accurately identify the position and degree of initial structural damage is of research and engineering significance to understanding structural operation states and evolutional trends, ensuring structural safety, and predicting structural behavior^2^.

Structural damage can induce changes in inherent characteristics of dynamic structural parameters, which further triggers changes in the dynamic response. Structural damage identification (SDI) issues utilize structural dynamic responses to realize inversion calculations of the location and degree of damage. The finite element model (FEM)-based correction method provides an effective approach to these issues. Its core idea is to achieve the minimum error between the analyzed benchmark FEM and dynamic and static load test results. By continuing to rectify FEM physical parameters, this method enables the FEM to more precisely characterize real structural response^3–10^. SDI can be therefore realized by comparing the structural parameters before and after damage. At present, correction methods of structural finite element model mainly use sensitivity analysis methods^11–15^ and a response surface method (RSM)^16–24^ to adjust the design parameters. There are multiple parameters including degree of freedom and correction parameters involved in correction of real structural FEM analyses. Sensitivity analysis methods show low efficiency as it requires to fulfil repeated iteration calculations of FEM; while RSM is a comprehensive experimental technology based on statistical analysis and mathematical modelling. It uses an approximate hypersurface to replace real structural eigenvalues and complex analytic relationship between parameters. The core idea of RSM is to approximate the relationship between structural input and output using a simple low order mathematical model by using experimental methods and numerical analysis. By doing so, RSM can realize the increase in correction efficiency through avoiding the use of FEM in each iteration^25^. A response surface-based FE model updating procedure for civil engineering structures in structural dynamics was proposed by Ren^16^. Shan D S A integrated a substructure-based finite element model updating technique with the correction method of RSM to propose a novel correction method of FEM for beam structures, which was applied in FEM correction of a composite cable-stayed bridge^17^. Ma et al. established correction methods of an RSM-based full-scale finite element model for concrete-filled steel tubular bridges, suspension bridges, and long-span continuous rigid frame bridges^18–24^. In recent years, as RSM-based FEM corrections have been increasingly reported, RSM still plays increasingly important roles in FEM correction in civil engineering, showing potential for development and research value. The method increases the amount of calculation in the process of selecting model parameters, thus reducing the computational burden of FEM analysis and improving the optimization efficiency and quality. Although RSM has been more widely applied in civil engineering, aerospace, and mechanical engineering, it is limited to the model correction and reliability analysis of structures, while it is rarely used for SDI.

The core part for RSM is the selection of response surface functions (RSFs). The commonly used RSFs include second-order polynomial, artificial neural network (ANN), support vector machine (SVM), and Kriging methods. Box and Wilson^26^ first proposed RSM, which is then developed and applied to the optimization design. Bucher^27^ proposed the second-order polynomial RSF without cross-terms and determined RSF parameters by using an interpolation method. Rajashekhar^28^ proposed a multi-step iterative RSM. The second-order polynomial RSF is simple, intuitive, and applicable in engineering practice. Despite these advantages, traditional optimization algorithms (least squares method, Newton’s method, and the Lagrange multiplier method) have shortcomings in terms of low calculation accuracy and poor convergence when solving second-order polynomial objective functions derived by RSM, so they cannot be applied in practice^29–31^. To solve the problem, Smith et al*.* introduced the ANN, SVM, and Kriging models to replace the second-order polynomial RSF^32–43^. Although the three RSFs (ANN, SVM, and Kriging) reduce errors, they also raise problems including the onerous computational burden, and low calculation efficiency. Due to these problems, the three RSFs cannot be readily used by engineers engaged in structural damage assessments, that is, they cannot be readily applied in practice. SDI can be transformed into non-linear optimization problems via RSM, namely, RSM needs to search for the optimal parameter solution of objective functions in a parameter design space. The smaller the objective function optimized, the smaller the error between theoretical models and reality. The FEM can more accurately characterize real structural mechanical behaviors. Thus, selecting a reasonable optimization algorithm is the key to tackling the problems about FEM corrections. Therefore, the imperialist competitive algorithm (ICA) is used to solve the second-order polynomial RSFs, aiming at the low accuracy and poor convergence of traditional optimization algorithms in solving second-order polynomial objective functions derived by RSM. This contributes to high calculation accuracy, good convergence, and high calculation efficiency of the second-order polynomial objective function. The RSM-ICA-based SDI method retains the simple and intuitional advantages of second-order polynomial RSFs and improves the calculation efficiency and accuracy of objective functions. Therefore, the method is more applicable to engineering and opens a new way for practical application of SDI.

The increasingly updating and alternating intelligent bionic optimization algorithms can overcome the limit of traditional algorithms. It can obtain abstract mathematical concepts by simulating the behavior of biological populations, which has been widely used in solving SDI problems in recent years^44–46^. The ICA^47^ proposed by Atashpaz-Gargari and Lucas in 2007 is a group random search optimization algorithm inspired by human social behaviors. It consists of initializing empires, colony assimilation, colony revolution, and competition among empires. ICA demonstrates good proximity search capability when solving the problems involving non-linear optimization; in addition, it has advantages including high efficiency in global search, flexible structure, and its rapid rate of convergence. Due to these benefits, its application in workshop task scheduling, path planning, fault diagnosis, and solving the travelling salesman problem has been broadly studied^48–51^. To solve the problem whereby incorporating the transportation times between the machines into the flexible job-shop scheduling arises, an adaptation of the ICA hybridized by a simulated annealing-based local search proposed by Karimi was presented^48^. Sadhu AK proposed a novel evolutionary optimization approach of solving a multi-robot stick-carrying problem-based ICA^49^. Zhang Yiyi developed a transformer fault diagnosis model that optimizes the SVM by the imperial colonial competition algorithm^50^. Pei Xiaobing proposed a hybrid ICA for solving issues concerning the travelling salesman problem combinatorial optimization^51^. However, little reach has been conducted into the application of ICA in solving SDI-related issues. ICA delivers a beneficial proximity search capability when dealing with non-linear optimization problems. Its application in real engineering can provide reference for structural micro-damage identification.

To find a novel idea for solving issues about structural parameter inversion, an RSM-ICA-based SDI method is proposed. This aims at the low accuracy and poor convergence of traditional optimization algorithms in solving second-order polynomial objective functions derived by RSM and attempts to provide a simple, accurate, and efficient damage identification method for practical SDI engineering. The damage identification principle and process of this new method are expounded in detail. The feasibility and reliability of the method have been verified. At first, a high-order response surface surrogate model with natural frequency response and stiffness reduction factor is constructed based on RSM. Then, objective functions are generated successively, furthermore, the ICA is used to optimize objective function parameters the further to identify the structural damage location and severity; lastly, the method is applied to the damage identification of a high-dimensional, locally-damaged, simply-supported beam numerical model to verify its reliability and feasibility. The first section presents an introduction; second section demonstrates the basic principle of RSM, the third section reveals the basic principle of ICA, the fourth section discusses the basic principle and application process of the RSM-ICA method; the fifth section presents the reliability and feasibility of RSM-ICA using numerical verification; the last section concludes.

## RSM

### The benchmark function of response surface and experimental design

The response surface benchmark function selected herein is an incomplete second-order polynomial without cross-terms, and expressed as

1 $$Z = a + \sum\limits_{i = 1}^{k} {b_{i} } x_{i} + \sum\limits_{i = 1}^{k} {c_{i} } x_{i}^{2}$$

where $x_{i}$, $x_{i}^{2}$ is a response surface benchmark function;$a$,$b_{i}$, and $c_{i}$ are undetermined coefficients; $k$ is the parameter vector; $k$ is the number of parameters (pcs).

Reliable experimental design plays a key role in solving problems involving the contradiction between ensuring the fitting precision when using RSM to substitute models and experimental cost. The number of samples is too small to reflect internal relationship between structural responses and parameters, causing reduction in fitting precision; the increase in number of sample points can improve precision of the model, while prolongs the cost of calculation and analysis. Therefore, it is important to select the finite and most representative sample data under the premise of without influencing computational precision.

The Latin hypercube design^52^ exhibits beneficial space-filling ability, which can reveal overall change using the limited number of samples, greatly improving sampling efficiency and precision. Hence, a stratified sampling based Latin hypercube design is adopted as an experimental design method.

### Response surface fitting and parameter significance analysis

Equation (1) can be expressed in a matrix form as

2 $${\varvec{Z}} = {\varvec{XK}}$$

In Eq. (2):

$${\varvec{X}} = \left[ {\begin{array}{*{20}c} 1 & {x_{11} } & {x_{12} } & \cdots & {x_{1k} } & {x_{11}^{2} } & {x_{12}^{2} } & \cdots & {x_{1k}^{2} } \\ 1 & {x_{21} } & {x_{22} } & \cdots & {x_{2k} } & {x_{21}^{2} } & {x_{22}^{2} } & \cdots & {x_{2k}^{2} } \\ \vdots & \vdots & \vdots & \ddots & \vdots & \vdots & \vdots & \vdots & \vdots \\ 1 & {x_{n1} } & {x_{n2} } & \cdots & {x_{nk} } & {x_{n1}^{2} } & {x_{n2}^{2} } & \cdots & {x_{nk}^{2} } \\ \end{array} } \right]$$ is the matrix of basis functions; $${\varvec{K}} = (\begin{array}{*{20}l} a \hfill & {b_{1} } \hfill & {b_{2} } \hfill & \cdots \hfill & {b_{k} } \hfill & {c_{1} } \hfill & {c_{2} } \hfill & \cdots \hfill & {c_{k} } \hfill \\ \end{array} )^{{\text{T}}}$$ is the vector of coefficients to be determined; *n* denotes the number of samples;$${\varvec{Z}} = (\begin{array}{*{20}c} {Z_{1} } & {Z_{2} } & {\begin{array}{*{20}c} \cdots & {Z_{n} } \\ \end{array} } \\ \end{array} )^{T}$$ is the response value vector.

According to the least squares principle, undetermined coefficient matrix is attained:

3 $$\user2{\rm K} = ({\varvec{X}}^{T} {\varvec{X}})^{ - 1} {\varvec{X}}^{T} {\varvec{Z}}$$

Based on stepwise regression analysis, the *F*-test method is used to perform the significance analysis of parameters. This method introduces or eliminates arbitrary parameters from response surface model, which is regarded as a stage (one part of a process) for stepwise regression. Each step should be subject to the *F* test. By doing so, that the model before introducing new parameters only contains items that significantly influence input values can be ensured. Through repeated introducing parameters, testing and elimination, optimal response surface can be acquired. It is assumed that the model contains *m* parameters, statistical variance is calculated to guarantee the significance of the (*m* + 1)th parameter.

4 $$F_{m + 1} = \frac{{(SSE_{m} - SSE_{m + 1} )/(\eta_{m} - \eta_{m + 1} )}}{{SSE_{m + 1} /\eta_{m + 1} }}$$

where,$SSE_{m}$ and $SSE_{m + 1}$ are the sum-of-squares error (SSE) for responses of the *m*th parameter and the (*m* + 1)th parameter in RSM; $\eta_{m}$ and $\eta_{m + 1}$ are the degree of freedom of the *m*th parameter and the (*m* + 1)th parameter*.*

Significance testing criteria of parameters are: in the case of given significance level $\alpha$, when $F_{m + 1} > F_{1 - \alpha } (1,n - m - 1)$, the significance of the (*m* + 1)th parameter is high, which needs to be introduced into RSM model, otherwise, will be eliminated.

### Precision testing of response surfaces

After establishing the RSM, whether there is such an approximate expression between structural response and parameters should be judged through analysis of variance to ensure that mathematical model can replace FEM to fulfil subsequent calculation and analysis of structural response values. The two error indices (multiple correlation coefficient $R^{2}$ and corrected multiple correlation coefficient $R_{Adj}^{2}$) can be used to test the predictive abilities of such response surfaces.

5 $$R^{2} = 1 - \frac{SSE}{{SST}} \, (0 \le R^{2} \le 1)$$

6 $$R_{Adj}^{2} = 1 - \frac{SSE/(n - m)}{{SST/(n - 1)}} \, (0 \le R_{Adj}^{2} \le 1)$$

7 $$SSE = \sum\limits_{i = 1}^{n} {(Z_{i} - \hat{Z}_{i} )^{2} }$$

8 $$SST = \sum\limits_{i = 1}^{n} {(Z_{i} - \overline{Z})^{2} }$$

In Eqs. (5) to (8), *SSE* denotes the sum of the squares of all deviations; *SST* is the total sum of squares of all deviations; $Z_{i}$ and $\hat{Z}_{i}$ refer to FEM response value and the response value of regression model for the *i*th sample point, with the average value $\overline{Z} = \frac{1}{n}\sum\limits_{i = 1}^{n} {Z_{i} }$.

## ICA

ICA is a novel meta-heuristic optimization algorithm that simulates the colonial competitive mechanism of empires in the development of human society. The evolutionary power of ICA mainly comes from constant competition among empires. The process of ICA includes: initializing empires, colony assimilation and revolution, and competitions and death of empire groups.

### Initializing empires

In the case of *k*-dimensional optimization, the location of a country is randomly assigned to each decisive variable to obtain a 1 × *k* matrix, we obtain:

9 $$Country = [p_{1} ,p_{2} , \ldots ,p_{k} ]$$

where $p_{1}$,$p_{2}$,…,$p_{k}$ are the parameters to be optimized, *k* is the number of parameters (pcs).

The cost function is used to derive the power of each country

10 $$Cost = f(Country) = f([p_{1} ,p_{2} , \ldots ,p_{k} ])$$

where $f( \cdot )$ is the cost function, the cost function is generally taken as the objective function of the optimization problem.

The cost functions of current countries are output and sequenced. The country with a small cost function is defined as $$N_{imp}$$ of the empire, other countries are defined as $$N_{col}$$, $$N_{pop} = N_{imp} +$$$$N_{col}$$ is the total number of countries (pcs). The colonies can be proportionally assigned. The standardizing cost of empires is processed thus:

11 $$C_{N} = c_{N} - \mathop {\max }\limits_{i} \{ c_{i} \}$$

where $c_{N}$ is the cost value of the *N*th empire; $\mathop {\max }\limits_{i} \{ c_{i} \}$ is the maximum cost among all empires..

The power of each empire compared to other empires can be expressed using the standardized cost:

12 $$p_{N} = \left| {\frac{{C_{N} }}{{\sum\nolimits_{i = 1}^{{N_{imp} }} {C_{i} } }}} \right|$$

where $p_{N}$ is the relative power of the Nth empire.

The colonies are divided among the empires according to their relative power, and the initial number of colonies controlled by the Nth empire is:

13 $$NC_{N} = round\{ p_{N} \cdot N_{col} \}$$

where round indicates rounding to ensure that the number of each colony is a whole number (pieces).

### Assimilation and revolutionary mechanism

**Figure 1:**
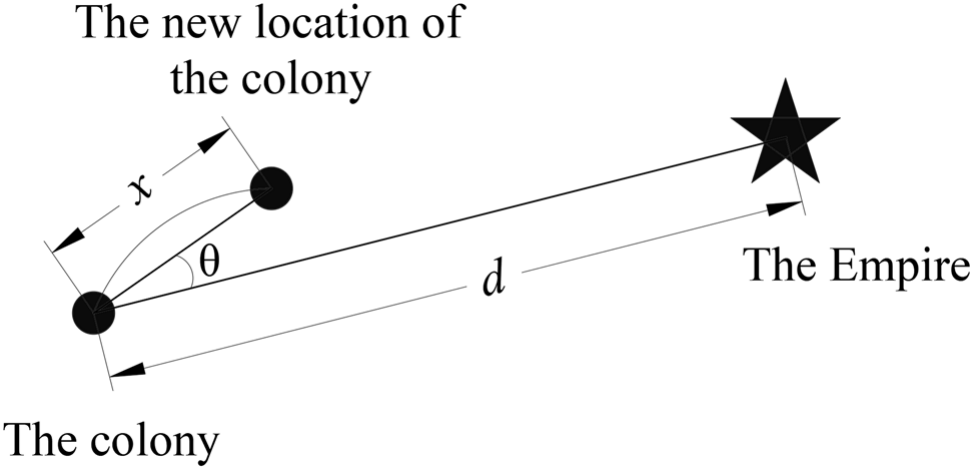
The moving path from the colonies to the empire.

The assimilation mechanism is defined as those empires attempt to absorb colonies under an empires’ control to become the part of the empire to enhance their imperial power. The process whereby colonies are constantly moving towards imperial movement, is as shown in Fig. 1; Assume that each movement of the colony towards the empire be a random number that obeys a uniform distribution $x \sim U(0,\beta \times d)$, $\beta (\beta > 1)$ denotes that the colony moves closer to the empire,generally $\beta = 2$, *d* is the distance between the colony and the empire. However, the colony does not necessarily move forward following the same direction of movement of the empire, thus a random angle subject to uniform distribution is introduced $\theta \sim U( - \sigma ,\sigma )$ to model this movement; where $\sigma$ is the parameter adjusting the deviation occurring between the parameter in ICA and original movement trace, generally $\sigma = \pi /4$.

The roles of the colony and the empire change dynamically. During the period when the colony moves towards the direction of the empire the cost of the colony is lower than the cost of empire, the empire and the colony will swap locations.

### Competition among different empire groups

In the competitive process, each empire group attempt to occupy the colonies of other empire groups to strengthen their power. The colonies of the empire group with weakest total imperial power will be carved up. The total cost of the imperial group consists mainly of its own costs and the costs of the colonies to which it belongs, expressed as, so the cost function of the total imperial power of an empire group is expressed as:

14 $$TC_{N} = c_{N} + \mu \frac{{\sum\nolimits_{i = 1}^{{NC_{N} }} {f(col_{Ni} )} }}{{NC_{N} }}$$

where $\mu$ is a weighting factor for the colonies' contribution to the power of the imperial Group, generally $\mu = 0.2$; $f(col_{Ni} )$ is the value of the cost of all colonies in the empire; ${{\sum\nolimits_{i = 1}^{{NC_{N} }} {f(col_{Ni} )} } \mathord{\left/ {\vphantom {{\sum\nolimits_{i = 1}^{{NC_{N} }} {f(col_{Ni} )} } {NC_{N} }}} \right. \kern-\nulldelimiterspace} {NC_{N} }}$ is the average of the cost of all colonies in the empire.

The total cost for empire standardization is determined as follows:

15 $$NTC_{N} = TC_{N} - \mathop {\max }\limits_{i} \{ TC_{i} \}$$

In Eq. (15): $\mathop {\max }\limits_{i} \{ TC_{i} \}$ is the maximum cost value among all empires. Then the relative power of each empire is:

16 $$p_{{p_{N} }} = \left| {\frac{{NTC_{N} }}{{\sum\nolimits_{i = 1}^{{N_{imp} }} {NTC_{i} } }}} \right|$$

The probability that an empire group occupies the weakest colony of the weakest empire group is

17 $${\varvec{D}} = [p_{{p_{1} }} - r_{1} ,p_{{p_{2} }} - r_{2} , \cdots ,p_{{p_{N} }} - r_{N} ]$$

In Eq. (17): $r_{1}$,$r_{2}$,…,$r_{N} \sim U(0,1)$ is a uniformly distributed random number.

In ***D***, The empire corresponding to the maximum value in D will occupy the weakest colony of the weakest empire.

### Empires perish

The empires with weaker power tend to gradually lose their colonies; as all colonies are gobbled up, empires perish and become a colony. Consequently, all colonies are ruled by only one empire, and the optimal solution can be deduced by algorithm convergence.

## SDI based on RSM-ICA

### Description of issues about SDI and mathematical model

The micro-structural damage changes the stiffness parameter. The FEM is corrected following the principle that parameters should match the real macro-response. By doing so, the stiffness parameter of damaged structures can be determined. Then, according to the location of parameter change and damage severity, the location, quantification, and identification of damage can be realized. SDI mathematical models can be transformed into optimized problems, and the optimal solution can be obtained using Eq. (18):

18 $$\left\{ \begin{gathered} {\text{Min }}\left\| {Z({\varvec{x}})} \right\| \, ^{2} , \, Z({\varvec{x}}) = \left\{ {Z_{e} } \right\} - \left\{ {Z_{c} } \right\} \hfill \\ s.t. \, VLB \le {\varvec{x}} \le VUB \hfill \\ \end{gathered} \right.$$

where ${\varvec{x}}$ is the vector of parameters to be corrected; $Z_{e}$ and $Z_{c}$ denote the structural model response and real structural response; $VLB$ and $VUB$ represent the upper and lower limits of the structural parameter; while $Z({\varvec{x}})$ is a residual error.

Statistic theory and modelling technique are integrated to construct a second-order polynomial RSM for structural responses and parameters. Thereafter, the established RSM is combined with the residual functions of structural model response and real structural response to achieve visual expression of the objective function.

### SDI process

**Figure 2:**
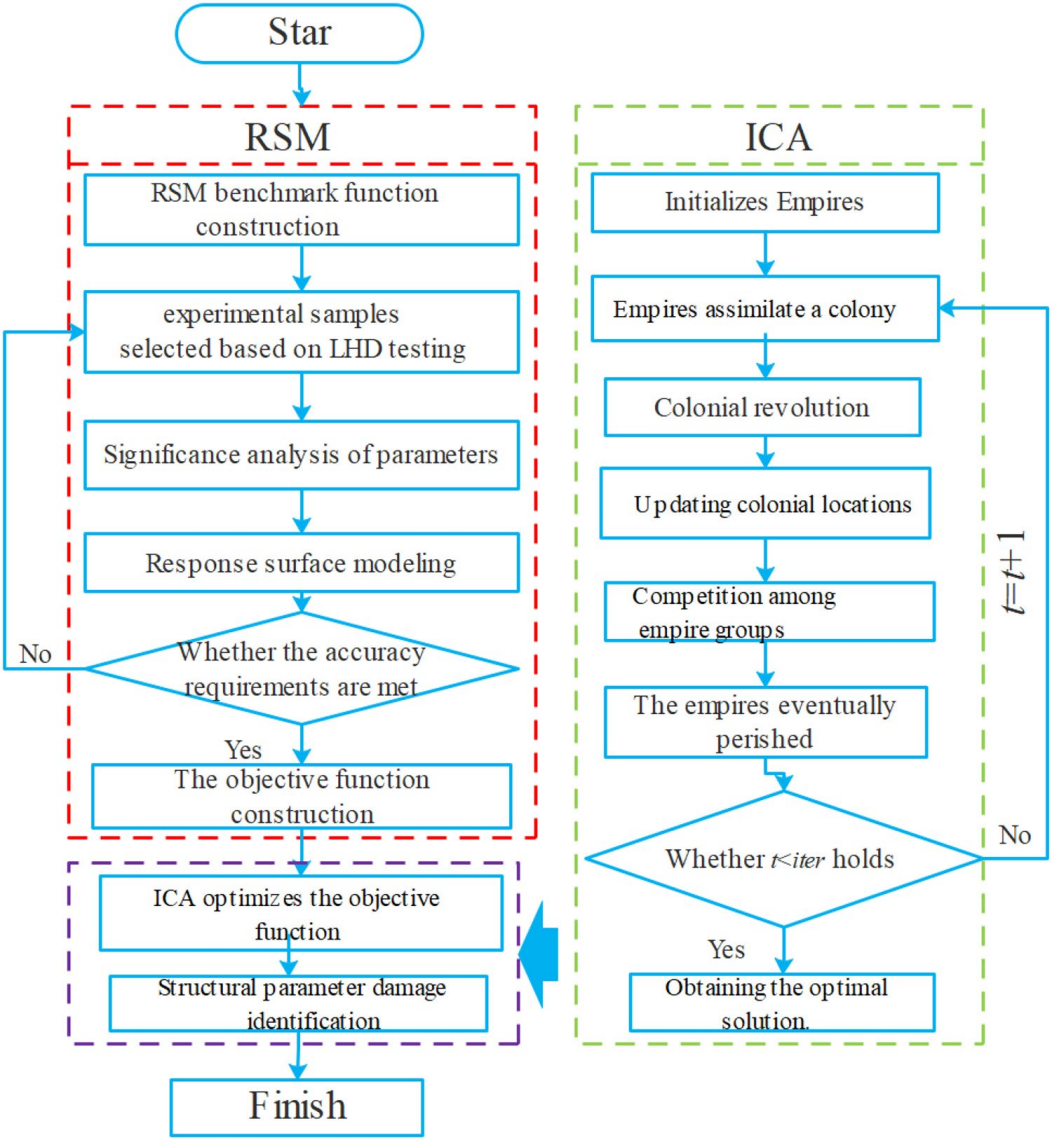
Flow chart of SDI based on RSM-ICA.

The flow chart of SDI based on RSM-ICA is demonstrated in Fig. 2.

The SDI process is described as follows:

1. RSM is used to determine the relationship between the structural stiffness parameter and an approximation thereto. On this basis, the residual functions of structural model response and real response can be generated as objective functions.
2. An ICA algorithm complied in MATLAB™ is run to optimize the parameters to be corrected, after obtaining the optimal corrected imperial location, the vector thereof is seen as the corrected parameter vector.
3. Comparing the changes in structural stiffness parameters before and after correction allows structural damage to be identified based on changes in the parameter location and damage severity.

## Numerical analysis

### Description of the simply-supported beam model

**Figure 3:**
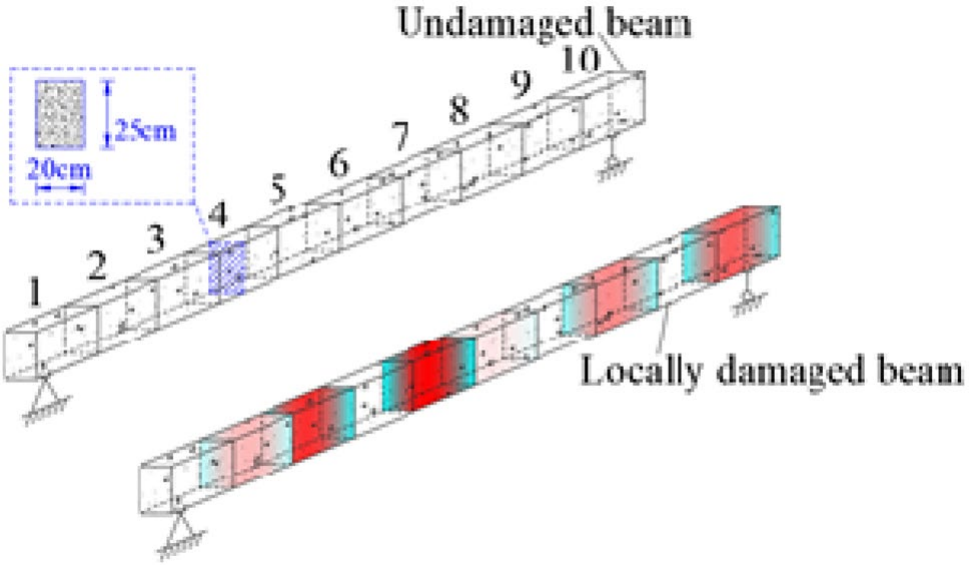
Numerical model of the simply supported beam.

Simply-supported beams are a form used in bridge structures that are widely applied to existing rural highways in China. Many such bridges were built in 1960s and 1970s designed with low bearing-capacity reserve. Combined with the increasing vehicle loads, damage has gradually developed in these bridges, so damage identification for such bridges is important to ensuring their safe operation. This research adopts a locally damaged simply-supported beam with a span of 6 m as a research object (Fig. 3). Based on RSM-ICA, the SDI of the beam is conducted to confirm the reliability and robustness of the proposed method. The cross-section of the beam measures 200 mm × 250 mm, the material density is set to 2 500 kg/m^3^, and material elastic modulus is 320 GPa. Furthermore, the benchmark FEM is established based on FEM and the structure of the beam is divided into 11 sections and 10 elements: because the structural damage of the beam is simulated considering the ways of stiffness reduction in some elements, the reduction of material elastic modulus is used to characterize stiffness reduction. Using an undamaged beam as the benchmark FEM model, it is assumed that damage occurs to Elements 2, 3, 5, 6, 8, and 10 in the beam, the stiffness of each element is reduced by 10%, 19%, 23%, 8%, 14%, and 15% respectively. The FEM model of this locally damaged beam is taken as representative.

Based on first five orders of natural frequencies of the FEM benchmark model and representative model, the maximum error of each natural frequency is 5.715% (Table 1). This implies that when a simply-supported beam with simple structure is subjected to local micro-damage, its natural frequency response deviates from that of the benchmark model. In consideration of this, the experimental data of a real model are adopted to correct the benchmark FEM. The corrected model is found to be able to match the mechanical behavior of real beam structures.

**Table 1:** Comparison of natural vibration frequencies between the benchmark and representative models.

Model	Frequency/Hz				
	First order	Second order	Third order	Fourth order	Fifth order
Benchmark model	11.133	20.098	44.245	54.933	98.398
Real model	10.519	19.151	42.104	52.492	92.775
Error/ %	5.515	4.710	4.839	4.443	5.715

Error = (benchmark frequency -real frequency)/ benchmark frequency × 100%

### Establishment of RSM-based objective function

The elastic modulus reduction coefficients *x* (real elastic modulus/benchmark elastic modulus) of 10 elements are treated as the parameters to be corrected, namely: the initial parameter vector of the undamaged simply-supported beam is [1.0, 1.0, 1.0, 1.0, 1.0, 1.0, 1.0, 1.0, 1.0, 1.0]^T^; the accurate corrected parameter vector of the locally damaged simply-supported beam is [1.0, 0.9, 0.81, 1.0, 0.77, 0.92, 1.0, 0.86, 1.0, 0.85]^T^. Given the stiffness of the actual beams in service is likely to weaken due to degradation of material properties and environmental erosion, the elastic modulus reduction coefficient of beam elements is set to [0.7, 1.0]. A Latin hypercube design is employed to perform experimental design by selecting 30 groups of sample point data, which are substituted into numerical models to calculate the first five orders of natural frequencies of each sample. All sample points and response values of Latin hypercube design are listed in Tables 2 and 3.

**Table 2:** Sample points for Latin hypercube design.

Sample no.	X1	X2	X3	X4	X5	X6	X7	X8	X9	X10
1	0.902	0.872	0.769	0.732	0.967	0.970	0.876	0.867	0.896	0.851
2	0.796	0.885	0.944	0.950	0.997	0.834	0.802	0.782	0.724	0.887
3	0.780	0.724	0.896	0.914	0.721	0.861	0.886	0.759	0.731	0.996
4	0.852	0.755	0.926	0.702	0.878	0.714	0.892	0.717	0.961	0.768
5	0.978	0.928	0.934	0.970	0.952	0.776	0.732	0.741	0.835	0.733
6	0.741	0.742	0.786	0.816	0.928	0.951	0.742	0.910	0.935	0.875
7	0.924	0.839	0.717	0.888	0.774	0.905	0.711	0.997	0.997	0.719
8	0.717	0.846	0.810	0.802	0.886	0.702	0.824	0.922	0.913	0.891
9	0.959	0.735	0.731	0.820	0.746	0.765	0.811	0.915	0.943	0.962
10	0.965	0.824	0.889	0.903	0.808	0.899	0.704	0.841	0.743	0.773
11	0.770	0.797	0.958	0.953	0.838	0.949	0.780	0.816	0.886	0.862
12	0.802	0.864	0.757	0.748	0.716	0.814	0.946	0.791	0.767	0.750
13	0.938	0.896	0.969	0.898	0.946	0.929	0.984	0.887	0.974	0.927
14	0.724	0.982	0.992	0.988	0.828	0.940	0.791	0.976	0.851	0.755
15	0.849	0.993	0.873	0.789	0.860	0.873	0.934	0.773	0.754	0.906
16	0.888	0.813	0.910	0.995	0.769	0.847	0.838	0.735	0.791	0.941
17	0.946	0.946	0.744	0.877	0.787	0.881	0.723	0.959	0.877	0.986
18	0.731	0.781	0.772	0.864	0.907	0.724	0.788	0.807	0.921	0.781
19	0.776	0.769	0.822	0.962	0.753	0.739	0.990	0.857	0.830	0.706
20	0.911	0.705	0.798	0.756	0.857	0.987	0.922	0.946	0.812	0.827
21	0.861	0.939	0.869	0.850	0.895	0.742	0.970	0.880	0.778	0.795
22	0.835	0.955	0.843	0.730	0.740	0.801	0.758	0.987	0.789	0.721
23	0.820	0.975	0.830	0.937	0.912	0.969	0.867	0.938	0.870	0.803
24	0.892	0.716	0.852	0.712	0.818	0.784	0.908	0.829	0.711	0.918
25	0.874	0.777	0.728	0.843	0.984	0.858	0.951	0.832	0.707	0.832
26	0.989	0.807	0.907	0.765	0.844	0.997	0.841	0.727	0.954	0.937
27	0.991	0.914	0.705	0.780	0.798	0.797	0.855	0.892	0.904	0.959
28	0.703	0.907	0.990	0.930	0.939	0.919	0.913	0.762	0.841	0.847
29	0.751	0.854	0.977	0.837	0.708	0.754	0.769	0.970	0.801	0.818
30	0.826	0.964	0.816	0.798	0.976	0.820	0.976	0.708	0.987	0.979

**Table 3:** Responses of the structure.

Sample no.	Z1/HZ	Z2/HZ	Z3/HZ	Z4/HZ	Z5/HZ
1	10.377	18.920	40.532	50.473	92.659
2	10.404	18.613	40.772	50.899	90.631
3	10.088	18.398	39.903	50.851	87.176
4	9.934	18.010	39.829	49.188	88.738
5	10.228	18.501	40.827	50.551	90.964
6	10.276	18.390	40.154	49.588	91.137
7	10.113	18.237	40.521	49.750	91.067
8	10.052	18.035	40.656	49.733	89.964
9	9.915	18.697	40.102	50.868	88.968
10	10.128	18.429	40.150	50.305	89.436
11	10.398	18.468	41.074	50.691	91.228
12	9.899	17.790	39.546	48.934	87.289
13	10.766	19.430	42.720	53.064	95.055
14	10.553	18.107	42.343	50.837	92.902
15	10.281	18.725	40.631	50.789	91.104
16	10.194	18.740	40.526	51.338	89.240
17	10.128	19.030	40.748	51.156	91.809
18	10.021	17.826	39.825	48.854	88.662
19	10.146	17.700	40.704	50.013	87.520
20	10.336	18.672	40.360	50.865	89.981
21	10.297	18.386	41.258	50.973	90.378
22	9.911	17.757	40.413	49.137	89.475
23	10.627	18.709	41.840	51.179	93.504
24	9.960	18.399	39.449	50.358	87.158
25	10.327	18.657	39.681	50.403	88.895
26	10.272	19.211	40.419	51.107	92.067
27	10.029	18.996	40.402	51.027	90.731
28	10.589	18.440	41.456	50.744	92.159
29	9.968	17.726	41.042	50.030	88.981
30	10.344	19.041	40.797	50.850	92.638

**Figure 4:**
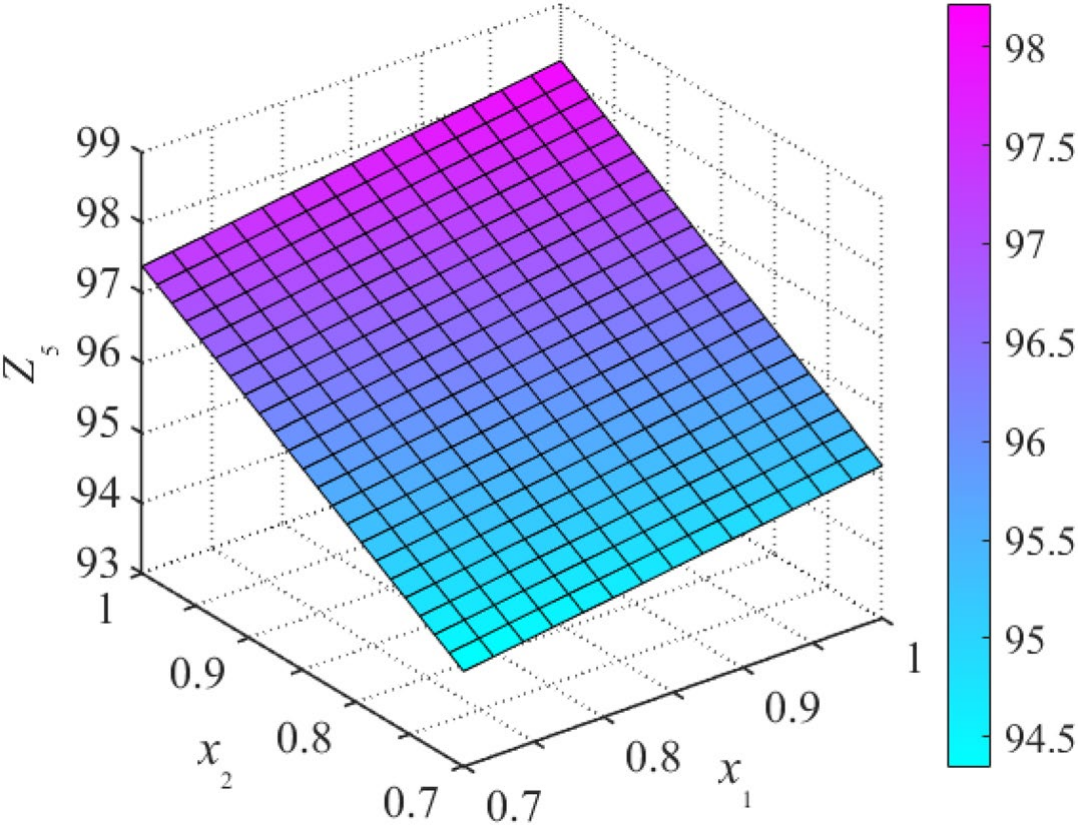
Response surfaces of frequency *Z*_5_ about *x*_1_ and *x*_2_.

**Figure 5:**
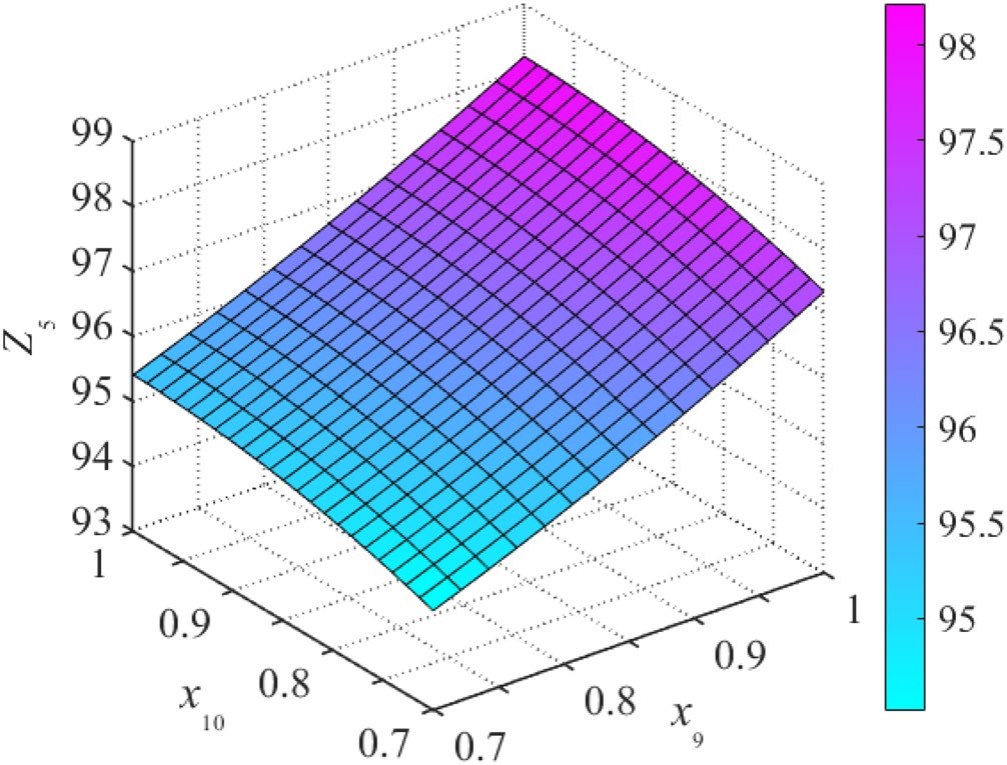
Response surface of frequency *Z*_5_ about *x*_9_ and *x*_10_.

Based on stepwise regression theory, the *F* test method is used to perform significance analysis of parameters. The optimal response surface can be obtained by removing the items of insignificance in the case without influencing fitting accuracy. An incomplete second-order polynomial is used to fit the datasets of sample points to obtain response surface Eqs. (19)–(23). According to the aforementioned polynomial, three-dimensional drawings of the structural natural frequency response surface, as illustrated in Figs. 4 and 5, the fitting performance of RSM is displayed in Table 4.

19 $$Z_{1} = 5.287 + 0.928x_{4} + 1.111x_{5} + 3.097x_{8} + 0.244x_{9} + 0.129x_{10} + 0.202x_{2}^{2} + 0.360x_{3}^{2} + 0.703x_{6}^{2} + 0.550x_{7}^{2} - {1}{\text{.477}}x_{8}^{2} \,$$

20 $$Z_{2} = 7.109 + 2.618x_{1} + 0.665x_{2} + 0.746x_{4} + 1.269x_{5} + 1.467x_{6} + 0.542x_{9} + 8.664x_{10} + 0.475x_{7}^{2} - 3.512x_{10}^{2}$$

21 $$Z_{3} = 24.028 + 0.713x_{1} + 3.357x_{2} + 3.185x_{4} - 12.924x_{6} + 17.294x_{8} + 2.653x_{9} + 0.769x_{10} + 2.696x_{3}^{2} + 0.406x_{5}^{2} + 8.008x_{6}^{2} + 1.709x_{7}^{2} - 7.613x_{8}^{2}$$

22 $$Z_{4} = 25.118 + 5.015x_{1} + 0.753x_{2} + 1.294x_{5} + 4.323x_{7} + 12.727x_{8} + 0.421x_{9} + 5.132x_{10} + 2.254x_{3}^{2} + 2.497x_{4}^{2} + 0.697x_{6}^{2} - 5.244x_{8}^{2}$$

23 $$Z_{5} = 39.061 + 2.764x_{1} + 10.094x_{2} + 6.773x_{5} + 1.357x_{7} + 28.824x_{8} + 17.986x_{10} + 2.959x_{3}^{2} + 1.075x_{4}^{2} + 4.604x_{6}^{2} - 13.935x_{8}^{2} + 5.500x_{9}^{2} - 8.853x_{10}^{2}$$

**Table 4:** Fitting effect of RSM.

RSM	*Z*_1_	*Z*_2_	*Z*_3_	*Z*_4_	*Z*_5_
$R^{2}$	0.998	0.999	0.997	0.997	0.999
$R_{Adj}^{2}$	0.997	0.998	0.995	0.995	0.998

As can be seen from Table 4, five types of multiple correlation coefficients and corrected coefficients all approximate to 1, indicating the overall goodness-of-fit and prediction ability of RSM within a certain parameter range. The precision obained is sufficient to replace FEM to perform subsequent calculation-based analysis.

### ICA-based on SDI

To establish the SDI objective function, SDI issues are converted into optimization issues based on Eq. (18):

24 $$\left\{ \begin{gathered} {\text{Min }}Z(x_{1} ,x_{2} , \ldots ,x_{10} ) \hfill \\ = {\text{Min [}}(Z_{1} - 10.519)^{2} + (Z_{2} - 19.151)^{2} + (Z_{3} - 42.104)^{2} \hfill \\ + (Z_{4} - 52.492)^{2} + (Z_{5} - 92.775)^{2} {]} \hfill \\ s.t. \, 0.7 \le x_{1} ,x_{2} , \ldots ,x_{10} \le 1.0 \hfill \\ \end{gathered} \right.$$

The ICA program compiled in MATLAB™ is used to derive optimal solution of parameters in Eq. (24) and the optimal stiffness reduction factor of elements $(x_{1} ,x_{2} , \ldots ,x_{10} )$ meeting the objective function is solved. By doing so, the error between the response of the benchmark model and damage model under a given incentive is minimized. The ICA parameters are listed in Table 5.

**Table 5:** Relevant parameters of ICA.

Parameters	Initial country size	The number of initial empires	Assimilation coefficient	Revolution rate	Success rate of revolution	Weight coefficient	Optimal dimension of parameterization	Upper limits of parameters	Lower limits of parameters	Maximum number of iterations
Values	50	10	1.5	0.5	0.1	0.2	10	1.0	0.7	100

**Figure 6:**
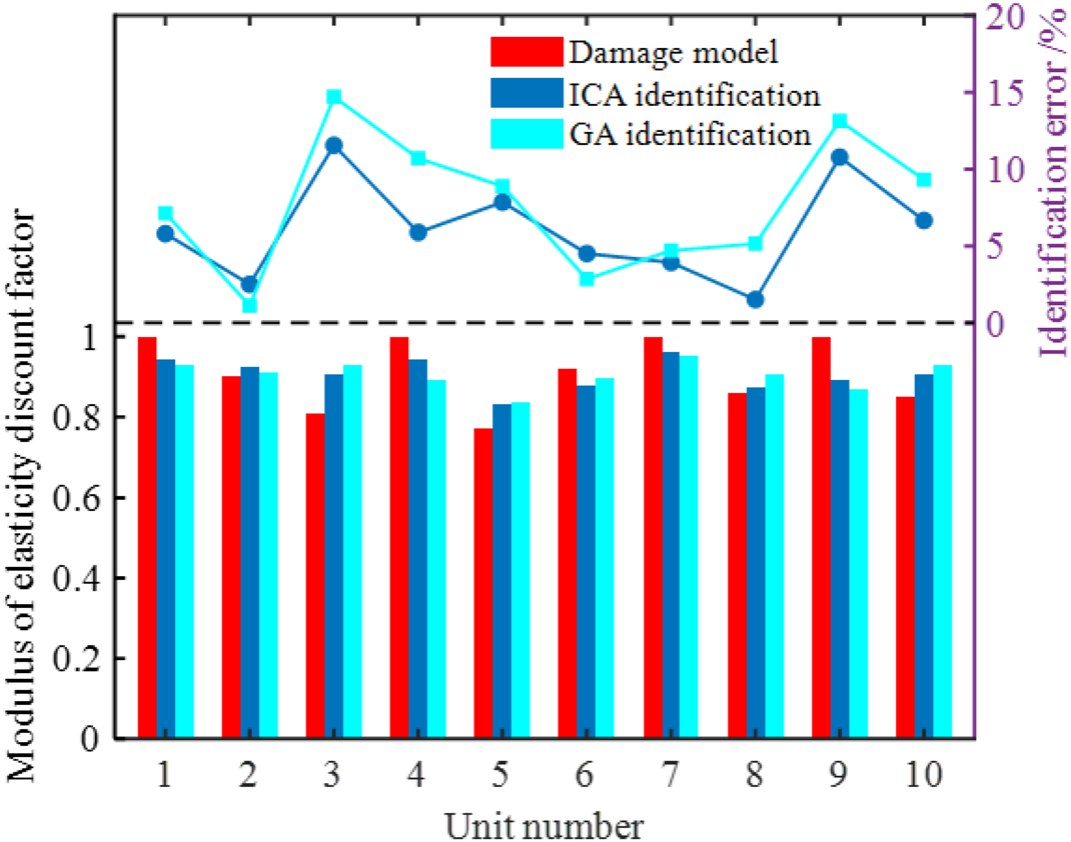
Comparison chart: SDI.

To eliminate the influence of the ergodicity of algorithm initialization, the average value of the optimized results over 20 cycles is taken as the optimal parameter vector. The identified results are compared with the results identified by genetic algorithm (GA) in MATLAB™ to validate the robustness and accuracy of the proposed method. The SDI results identified based on ICA and GA are displayed in Tables 6 and Fig. 6.

**Table 6:** Comparison of damage identification results.

Description	Damage value	RSM-ICA				RSM-GA		
		Identified values	Identification error/%		Mean error/%	Identified values	Identified error/%	Mean error/%
Elastic modulus reduction factor	*X*_1_	1.000	0.942	5.806	6.104	0.929	7.140	7.774
	*X*_2_	0.900	0.923	2.516		0.910	1.129	
	*X*_3_	0.810	0.904	11.552		0.929	14.693	
	*X*_4_	1.000	0.941	5.891		0.893	10.704	
	*X*_5_	0.770	0.830	7.854		0.838	8.891	
	*X*_6_	0.920	0.878	4.513		0.894	2.853	
	*X*_7_	1.000	0.961	3.933		0.953	4.709	
	*X*_8_	0.860	0.873	1.521		0.904	5.155	
	*X*_9_	1.000	0.892	10.783		0.869	13.143	
	*X*_10_	0.850	0.907	6.668		0.929	9.318	
Natural frequency	*Z*_1_	10.519	10.532	0.121	0.134	10.535	0.155	0.175
	*Z*_2_	19.151	19.128	0.123		19.134	0.090	
	*Z*_3_	42.104	42.165	0.143		42.169	0.155	
	*Z*_4_	52.492	52.567	0.144		52.608	0.221	
	*Z*_5_	92.775	92.905	0.140		93.010	0.254	

Identification error = (damage value – identified value)/damage value × 100%

As can be seen from Table 6 and Fig. 6, the comparison of elastic modulus reduction coefficient and damage values of different sections identified using different algorithms shows that the identification accuracy based on ICA is satisfactory with the maximum and mean errors of 11.552% and 6.104% separately in parameter identification. In comparison, the maximum and mean errors of parameter identification based on GA are separately 14.693% and 7.774%. Except for damage parameters in Elements 2 and 6, the parameter identification errors based on ICA for other elements are significantly lower than those of the GA-based method. The elastic modulus reduction coefficient in Element 5 after identification is only 0.830, which is the minimum among all identification results, indicating the occurrence of a high degree of structural damage at that position.

Mean errors of the first five orders of natural frequencies of the structures identified using the two methods are 0.134% and 0.175%, respectively. The identification accuracy of ICA improves significantly. Except for the second-order natural frequency of simply-supported beams, errors in the other four natural frequencies all reduce to a significant extent. The ICA-based SDI method has higher accuracy and efficiency than the GA-based SDI method. The natural frequency of each order for simply-supported beams identified using the proposed ICA-based method has an error between 0.121% and 0.144% with the natural frequency of damage models, indicating a good match in terms of modeled coincidence. The parameters identified can thus match actual macro-responses of a damaged structure.

## Conclusion

In this research, an RSM-ICA-based SDI method and its application are elaborated. Local damage to simply-supported beams is taken as a numerical example to validate the application feasibility of this method in solving high-dimensional problems. The main conclusions include:

1. In the inversion process of structural damage problems, the polynomial function between the elastic modulus reduction coefficient and structural responses is constructed based on RSM and the FEM of structures is replaced with RSM. This avoids repeated calculations of FEM in the SDI process, which improves SDI efficiency.
2. The RSM-ICA-based SDI method shows a distinct calculation process and is capable of accurately identifying the material parameters of the micro-damage model. It can locate and quantify damaged zones. This identification method only needs to consider the first few natural frequencies of structures. Errors of identified structural parameter and response are 6.104% and 0.134%, showing a more significant improvement in accuracy compared with conventional algorithms. This method provides an accurate and efficient SDI technology.
3. The RSM-ICA-based SDI method can be used in micro-damage identification on large-scale complex bridge structures. It is expected to enable bridge health monitoring, bridge safety evaluation, and damage prediction to realize precision development by deeply fusing SDI and intelligent algorithms.

## Data Availability

All data generated or analyzed during this study are included in this published article.
